# Understanding perception of active noise control system through multichannel EEG analysis

**DOI:** 10.1049/htl.2017.0016

**Published:** 2018-05-08

**Authors:** Sangeeta Bagha, R.K. Tripathy, Pranati Nanda, C. Preetam, Debi Prasad Das

**Affiliations:** 1 Department of Process Modelling and Instrumentation, CSIR-Institute of Minerals and Materials Technology, Bhubaneswar, India; 2 Academy of Scientific and Innovative Research (AcSIR), India; 3 Silicon Institute of Technology, Bhubaneswar, India; 4 Faculty of Engineering and Technology (ITER), Siksha ‘O’ Anusandhan, Bhubaneswar, India; 5 Department of Physiology, All India Institute of Medical Sciences (AIIMS), Bhubaneswar, India; 6 Department of ENT, All India Institute of Medical Sciences (AIIMS), Bhubaneswar, India

**Keywords:** electroencephalography, active noise control, discrete wavelet transforms, singular value decomposition, medical signal processing, signal classification, active noise control system, multichannel EEG analysis, ANC, electroencephalogram, silent listening condition, music, background noise, multiscale analysis, discrete wavelet transform, multivariate multiscale matrices, sub-band signals, singular value decomposition, multivariate matrices, singular value features, extreme learning machine classifier, activation functions, human brain

## Abstract

In this Letter, a method is proposed to investigate the effect of noise with and without active noise control (ANC) on multichannel electroencephalogram (EEG) signal. The multichannel EEG signal is recorded during different listening conditions such as silent, music, noise, ANC with background noise and ANC with both background noise and music. The multiscale analysis of EEG signal of each channel is performed using the discrete wavelet transform. The multivariate multiscale matrices are formulated based on the sub-band signals of each EEG channel. The singular value decomposition is applied to the multivariate matrices of multichannel EEG at significant scales. The singular value features at significant scales and the extreme learning machine classifier with three different activation functions are used for classification of multichannel EEG signal. The experimental results demonstrate that, for ANC with noise and ANC with noise and music classes, the proposed method has sensitivity values of 75.831% ($p \lt 0.001$) and 99.31% ($p \lt 0.001$), respectively. The method has an accuracy value of 83.22% for the classification of EEG signal with music and ANC with music as stimuli. The important finding of this study is that by the introduction of ANC, music can be better perceived by the human brain.

## 1 Introduction

Acoustic noise is increasing day-by-day due to the excessive use of machinery. The perception of noise by a human being is an important research area [1]. There is an increasing concern for noise reduction with additional cost [2]. Noise-induced stress in children has been studied [3]. Stress is related to brain activity and hence researchers study the stress using electroencephalogram (EEG) under different listening conditions such as noise and music [4]. Music is liked by a person and may reduce stress, whereas noise is not liked and may induce stress. Multichannel EEG provides the spatial and the temporal information about the brain electrical activity and it is widely used for detection of Alzheimer disease [5], epileptic seizures [6, 7], sleep apnea [8] and different types of emotions [9, 10]. The EEG signal consists of the clinical patterns such as $\delta $, $\theta $, $\alpha $ and $\beta $ waves of different frequency ranges. The frequency ranges of these patterns ($\delta $, $\theta $, $\alpha $ and $\beta $ waves) are [0.1–3.9 Hz], [4–7.9 Hz], [8–13.9 Hz] and [14–30 Hz], respectively [11]. The $\delta $ wave is present during dreamless sleep and it has the highest amplitude. The $\theta $ wave is associated with learning and memory and it is present in dreaming sleep. Similarly, during drowsy, relaxation and meditative stages, the $\alpha $ waves appear in the EEG signal. The $\beta $ wave is the high-frequency component of the EEG signal and it is present during alertness, anxiety, fear and stress [12]. The study on the effect of sound listening on human EEG is limited, unlike noise. Different sounds have different effects on the human brain as they have a different range of noise frequency and power. Quantifying the changes in the EEG signal during different listening conditions is a challenging problem.

In the literature, various methods have been proposed like time–frequency analysis [13, 14], wavelet analysis [15], detrained fluctuation analysis (DFA) [16], multifractal DFA [17] for quantifying the physiological changes in the EEG signal during external stimuli such as the noise, the listening of music and the induced emotion [18]. Lin *et al.* [19] have used independent component analysis to differentiate the gamma band synchrony between musicians and non-musicians. Listening to music can change the brain chemistry [20] and decrease the pain and anxiety in critically ill patients by activating pleasure-seeking areas of the brain [21]. The state-of-art methods for the analysis of EEG signal using music as a stimulus are shown in Table 1. The emotional excitation of the EEG of different regions of the brain varies depending on the types of music stimuli [22]. It has also been found that the pink noise exposure can induce a stable sleep stage [4]. The left frontal activity of the brain increases with positive emotion, whereas the corresponding right frontal activity increases with negative emotion in response to music [23]. The theta activation in frontal-midline, T3, and Pz electrodes can vary with music-induced emotions [24]. However, the effect of active noise control (ANC) on brain electrical activity is entirely a new study and paves the way for the future research.

**Table 1:** State-of-art of methods for analysis of EEG signal with music as stimulus

Authors	Signal processing techniques used	Classes of EEG signals with music as stimulus
Hadjidimitriou and Hadjileontiadis [14]	time–frequency analysis and KNN classifier	like versus dislike to music stimulus
Hadjidimitriou and Hadjileontiadis [13]	time–frequency analysis, and familiarity ratings	like versus dislike to music stimulus
Sturm *et al.* [25]	spatio-temporal regression filters and SVD	naturalistic music stimulus
Bhoria *et al.* [26]	bandpass filtering and power spectral density-based analysis of EEG	no music, music with 60 dB sound level, music with 75 dB sound level and music with 100 dB sound level
Georgescu *et al.* [27]	statistical analysis of alpha, beta, delta and theta bands	monotonous auditory stimulation

ANC works on the principle of superposition where a secondary noise of the same amplitude and opposite phase of the primary noise at the appropriate spatial location is generated by the controller to cancel the noise in the acoustic medium [28]. The ANC is very effective in controlling the low-frequency noise generated by the air-conditioning system, rotating fan, ventilators, incubators and so on, which is more annoying to the ear than the broadband noise of the same dB like speech and music. The beeping sound of various medical equipment in the intensive care unit of hospitals gives irritating experience to the patients. Continuous exposure to tonal/beeping noise can adversely affect the auditory system, increase stress, and induce physiological problems [1]. In this Letter, we have investigated the effect of ANC mechanism on EEG signal. The discrete wavelet transform (DWT) decomposes the EEG signal into a vector of wavelet coefficients at different sub-bands [29]. The wavelet coefficients of these scales capture the information about the $\delta $, $\theta $, $\alpha $ and $\beta $ waves. It is expected that the effect of ANC on the brain electrical activity can be effectively studied using the features of EEG signal at different wavelet scales. In this work, the multiscale eigenspace analysis of multichannel EEG signal is performed. The singular value features from the significant sub-band matrices of multichannel EEG signal are extracted and these features are given to extreme learning machine (ELM) model for classification of silent, noise, music, ANC with noise and ANC with noise and music. The effect of ANC on the features of multichannel EEG is verified through the statistical analysis and the performance of ELM classifier. The remainder of this letter is arranged as follows. In Section 2, the method for investigating the effect of ANC to brain electrical activity is described. The results and the discussion are presented in Section 3 and in Section 4, the conclusion of this letter is drawn.

## 2 Proposed method

**Fig. 1:**
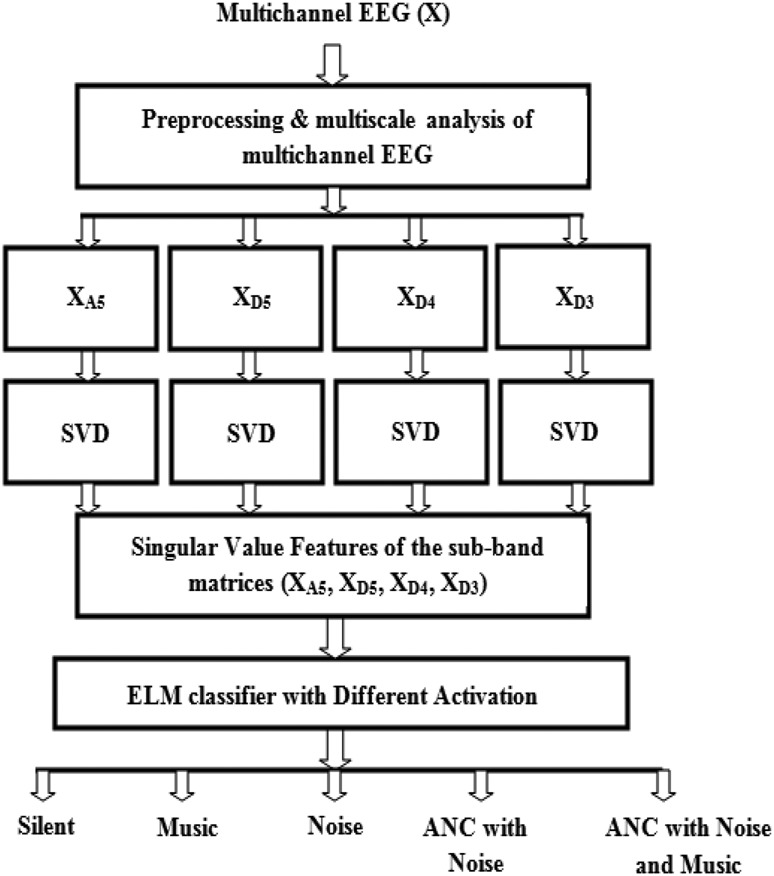
Block diagram for analysis and classification of multichannel EEG signals

The block diagram of the proposed method for investigating the effect of ANC mechanism on EEG signal is shown in Fig. 1. It consists of six stages, such as multichannel EEG database creation, preprocessing and multiscale analysis of multichannel EEG, sub-band matrix formulation, eigenanalysis of the sub-band matrices at significant scales using singular value decomposition (SVD), eigenvalue feature selection and classification. The details of each block are explained in the following subsections.

### 2.1 EEG database creation

The recording of EEG signal is done using a 24/32 channel EEG recorder (RMS Maximus Electroencephalograph). The measurement electrodes (FZ, CZ, PZ, FP1, FP2, F3, F4, C3, C4, P3, P4, O1, O2, F7, F8, T3, T4, T5 and T6) and reference electrodes (A1 and A2) are placed at the appropriate position of the individual subject with conductive paste. The sampling rate of each channel EEG signal is 256 Hz. Multichannel EEG signals are obtained from three subjects (mean age 33, three males). All the subjects have undergone audiometry test and reported normal hearing. Here, we have recorded the EEG in five different conditions. First, the EEG is recorded for 7 min when the subject is in the relaxed but awake state with eyes closed. At that time, there is low background sound. Then, the subject is made to listen to music of his interest for 7 min and the EEG signal is recorded. The subject is also exposed to a recorded broadband noise for 7 min and the corresponding EEG signal is recorded. Then, the subject wore an ANC headphone (BOSE ANC headphone) and exposed to the same broadband noise for another 7 min and the resulting multichannel EEG data is collected. After that, the same condition is continued with music played through the BOSE ANC headphone for another 7 min.

### 2.2 Preprocessing

The preprocessing includes the filtering of noises from the multichannel EEG signal and the segmentation of multichannel EEG data into frames. The filtering is done during the recording of multichannel EEG data using RMS Maximus Electroencephalograph. The low-pass filter (cut-off frequency as 75 Hz) is considered to eliminate the high-frequency artefacts from each channel EEG signal [11]. Similarly, to filter out the baseline wandering noise, a high-pass filter (cut-off frequency as 0.5 Hz) has been used [11]. After filtering, each channel EEG is divided into frames of 512 samples. We have considered 3000 number of multichannel EEG frames with each of size $512 \times 19$ for feature extraction. The rows and columns indicate samples and channels of the EEG, respectively. The frame-based processing of multichannel EEG data is considered to capture both spatial and temporal correlations.

### 2.3 Multiscale eigenanalysis of multichannel EEG

The multiscale analysis using DWT has the advantage to capture the clinical patterns of the EEG signal in different scales [29]. The wavelet coefficients of multichannel EEG at approximation and detail scales are evaluated using the inner product of each channel EEG data with scaling function and wavelet function [30]. The scaling and the wavelet functions are computed based on the dilation and the translation of the mother wavelet. In this Letter, we have used ‘db4’ mother wavelet. The scaling and the wavelet functions are given by $\phi _{l\comma k}\lpar n\rpar = 2^{ - l/2}\phi \lpar 2^{ - l}n - k\rpar $ and $\psi _{l\comma k}\lpar n\rpar = 2^{ - l/2}\psi \lpar 2^{ - l}n - k\rpar $, respectively. The wavelet coefficients for the *p*th channel EEG frame in approximation and detail sub-bands are evaluated as $cA_L^p \lpar k\rpar = \left\langle {x^p\lpar n\rpar \comma \; \phi _{L\comma k}\lpar n\rpar } \right\rangle $ and $cD_l^p \lpar k\rpar = \left\langle {{\bi x}^p\lpar n\rpar \comma \psi _{l\comma k}\lpar n\rpar } \right\rangle $, respectively, where *L* is the decomposition level and $l = 1\comma \; 2\comma \; \ldots \comma \; L$. The wavelet coefficients can be evaluated based on the filter bank realisation where the scaling functions and the wavelet functions are termed as low-pass and high-pass filters, respectively [31]. The signal reconstructed using only one sub-band wavelet coefficients is called as the sub-band signal. The approximation and the detail sub-band signals for the *p*th channel are evaluated as ${\bi x}_{AL}^p = \left\langle {cA_L^p \lpar k\rpar \comma \; {\tilde \phi }_{L\comma k}\lpar n\rpar } \right\rangle $ and ${\bi x}_{Dl}^p = \left\langle {cD_l^p \lpar k\rpar \comma \; {\tilde \psi }_{l\comma k}\lpar n\rpar } \right\rangle $, respectively [31], where $\tilde \phi _{L\comma k}\lpar n\rpar $ and $\tilde \psi _{l\comma k}\lpar n\rpar $ are the reconstruction filters for low-pass and high-pass sections, respectively. The approximation and the detail sub-band matrices are formulated using the sub-band signal of the *p*th channel and these are given as

(1) $${\bi X}_{AL} = \lsqb {\bi x}_{AL}^1 \comma \; {\bi x}_{AL}^2 \comma \; \ldots \comma \; {\bi x}_{AL}^p \rsqb \eqno\lpar 1\rpar $$

(2) $${\bi X}_{Dl} = \lsqb {\bi x}_{Dl}^1 \comma \; {\bi x}_{Dl}^2 \comma \; \ldots \comma \; {\bi x}_{Dl}^p \rsqb \eqno\lpar 2\rpar $$

**Fig. 2:**
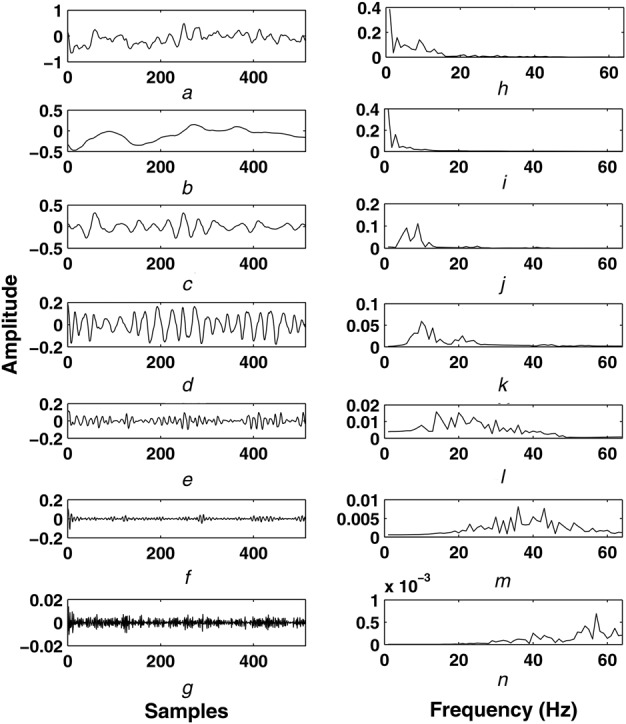
Multiresolution analysis of EEG Signal using DWT *a* EEG signal of Fp1 channel *b* $cA_5$ sub-band signal *c* $cD_5$ sub-band signal *d* $cD_4$ sub-band signal *e* $cD_3$ sub-band signal *f* $cD_2$ sub-band signal *g* $cD_1$ sub-band signal *h* Spectrum of EEG signal *i* Spectrum of $cA_5$ sub-band signal *j* Spectrum of $cD_5$ sub-band signal *k* Spectrum of $cD_4$ sub-band signal *l* Spectrum of $cD_3$ sub-band signal *m* Spectrum of $cD_2$ sub-band signal *n* Spectrum of $cD_1$ sub-band signal

In this work, the number of decomposition levels is chosen as five for each EEG channel. Six sub-band matrices are formulated for each multichannel EEG frame. Out of these sub-band matrices, ${\bi X}_{D5}$, ${\bi X}_{D4}$, ${\bi X}_{D3}$, ${\bi X}_{D2}$ and ${\bi X}_{D1}$ are the detail sub-band matrices and ${\bi X}_{A5}$ is the approximation sub-band matrix. The sub-band matrices of multichannel EEG are selected based on the frequency content. The EEG signal for Fp1 channel and the sub-band signals are shown in Figs. 2*a*–*g*, respectively. The spectra of the $cA_5$, $cD_5$, $cD_4$, $cD_3$, $cD_2$ and $cD_1$ sub-band signals are shown in Figs. 2*i*–*n*, respectively. The frequency ranges of $cA_5$, $cD_5$, $cD_4$, $cD_3$, $cD_2$ and $cD_1$ sub-band signals are given as [0–4 Hz], [4–8 Hz], [8–16 Hz], [16–32 Hz], [32–64 Hz] and [64–128 Hz], respectively [32]. The bandwidth of EEG signal in this work is considered as [0.5–75 Hz]. It is evident that, the $\delta $-wave information is captured using $cA_5$ sub-band signal, whereas the $cD_5$ sub-band signal contains the $\theta $-wave information. Similarly, the $\alpha $-wave and $\beta $-wave information is captured using the $cD_4$ and $cD_3$ sub-band signals, respectively. These four sub-band signals of each channel EEG frame are considered for eigenanalysis.

The sub-band matrices ${\bi X}_{A5}$, ${\bi X}_{D5}$, ${\bi X}_{D4}$ and ${\bi X}_{D3}$ capture the information of multichannel EEG signal. The eigenanalysis of these four matrices are performed using SVD [33]. The SVD of the approximation and the detail sub-band matrices are given as

(3) $${\bi X}_{AL} = {\bi U}_{AL}{\bf \Lambda }_{AL}{\bi V}_{AL}^{\rm T} \eqno\lpar 3\rpar $$

(4) $${\bi X}_{Dl} = {\bi U}_{Dl}{\bf \Lambda }_{Dl}{\bi V}_{Dl}^{\rm T} \comma \; \quad l = 1\comma \; 2\comma \; 3\comma \; 4\comma \; 5\eqno\lpar 4\rpar $$

where ${\bi U}_{AL}$ and ${\bi V}_{AL}$ are the left eigenmatrix and the right eigenmatrix for approximation sub-band matrix. Similarly, the left eigenmatrix and the right eigenmatrix for the *l*th detail sub-band matrix are ${\bi U}_{Dl}$ and ${\bi V}_{Dl}$, respectively [33]. $\sigma _{AL1}\comma \; \sigma _{AL2}\comma \; \ldots \comma \; \sigma _{ALp} = {\rm diag}\lpar {\bf \Lambda }_{AL}\rpar $ and $\sigma _{1Dl}\comma \; \sigma _{2Dl}\comma \; \ldots \comma \; \sigma _{\,pDl} = {\rm diag}\lpar {\bf \Lambda }_{Dl}\rpar $ are the singular value matrices for approximation sub-band matrix and the *l*th detail sub-band matrix, respectively. In this Letter, the singular values of ${\bi X}_{A5}$, ${\bi X}_{D5}$, ${\bi X}_{D4}$ and ${\bi X}_{D3}$ sub-band matrices are used as features for classification.

### 2.4 Feature selection and classification

In this work, the first eight singular values from each sub-band matrix of multichannel EEG are used. The first eight singular value features of ${\bi X}_{A5}$ sub-band matrix are termed as the delta-band features, as it captures the information of $\delta $ wave. Similarly, the first eight singular values of ${\bi X}_{D5}$, ${\bi X}_{D4}$ and ${\bi X}_{D3}$ matrices are called as the theta-band, the alpha-band and the beta-band features. The singular value features of ${\bi X}_{A5}$, ${\bi X}_{D5}$, ${\bi X}_{D4}$ and ${\bi X}_{D3}$ sub-band matrices are combined to formulate a 32-dimensional (32D) feature vector. The 32D feature vector, 8D delta-band feature vector, 8D theta-band feature vector, 8D alpha-band feature vector and 8D beta-band feature vector are used as input to the ELM classifier [34]. The feature matrix (${\bi Z} \in R^{n \times m}$) used in this work consists of a *n* number of instances or multichannel EEG frames and *m* number of features. The ELM classifies the feature vector of multichannel EEG into silent, noise, music, ANC with noise and ANC with music and noise classes. The class labels for silent, noise, music, ANC with noise and ANC with music with noise classes are assigned as *y*_1_ (00001), *y*_2_ (00010), *y*_3_ (00100), *y*_4_ (01000) and *y*_5_ (10000), respectively.

The sensitivity (SE) of the $i{\rm th}$ class ($i = 1\comma \; 2\comma \; 3\comma \; 4\comma \; 5$) is evaluated as

(5) $${\rm S}{\rm E}_i = \displaystyle{{a_{ii}} \over {\sum\nolimits_{\,j = 1}^5 {a_{ij}} }} \times 100\eqno\lpar 5\rpar $$

Similarly, the overall accuracy (OA) of the ELM classifier is given as, ${\rm OA} = \left({\sum\nolimits_{i = 1}^5 {\rm S}{\rm E}_i/5} \right)$.

In this Letter, we have used the 5-fold cross-validation approach for selecting the training and test EEG feature instances of the ELM classifier. The ELM is a single-layer feedforward neural network in which the input feature vector is mapped to the hidden layer space or ELM feature space [34]. The weights between the hidden layer and the output layer in ELM are evaluated by solving the least square regularisation problem and the hidden neurons are randomly assigned [35]. For multiclass classification using ELM, the output layer has a *q* number of neurons. Here, we have considered the number of output neurons as $q = 5$ for ELM. The activation functions such as ‘sigmoid’, ‘sine’ and radial basis function (‘radbas’) are used [35]. The performance of ELM classifier on EEG features is compared using these activation functions. The sensitivity and the overall accuracy measures are considered to quantify the performance of ELM classifier and these measures are evaluated from the confusion matrix [36]. The confusion matrix for the five class ELM classifiers is given in Table 2.

**Table 2:** Confusion matrix for the proposed five class classification task

	Predicted					
A		*y*_1_	*y*_2_	*y*_3_	*y*_4_	*y*_5_
C	*y*_1_	$a_{11}$	$a_{12}$	$a_{13}$	$a_{14}$	$a_{15}$
T	*y*_2_	$a_{21}$	$a_{22}$	$a_{23}$	$a_{24}$	$a_{25}$
U	*y*_3_	$a_{31}$	$a_{32}$	$a_{33}$	$a_{34}$	$a_{35}$
A	*y*_4_	$a_{41}$	$a_{42}$	$a_{43}$	$a_{44}$	$a_{45}$
L	*y*_5_	$a_{51}$	$a_{52}$	$a_{53}$	$a_{54}$	$a_{55}$

## 3 Results and discussion

**Fig. 3:**
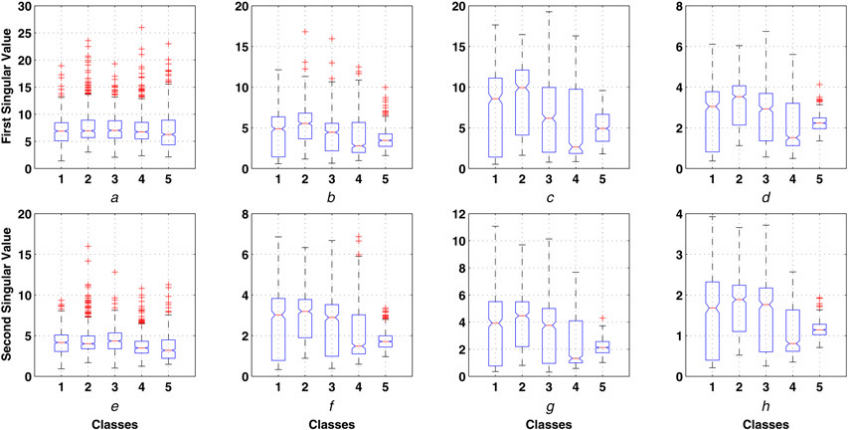
Intra-class variations of first singular value feature of *a* ${\bi X}_{{\bi A5}}$ sub-band matrix *b* ${\bi X}_{{\bi D5}}$ sub-band matrix *c* ${\bi X}_{{\bi D4}}$ sub-band matrix *d* ${\bi X}_{{\bi D3}}$ sub-band matrix *e* ${\bi X}_{{\bi A6}}$ sub-band matrix *f* ${\bi X}_{{\bi D5}}$ sub-band matrix *g* ${\bi X}_{{\bi D4}}$ sub-band matrix *h* ${\bi X}_{{\bi D3}}$ sub-band matrix (classes: 1 – silent, 2 -noise, 3-music, 4-ANC with noise, 5- ANC with music and noise)

In this section, the statistical analysis of the proposed singular value features of sub-band matrices of multichannel EEG and the performance of ELM classifier are shown. The statistical analysis is performed based on the evaluation of the mean and the standard deviation values of each class. The within-class variations of the first and the second singular value features of ${\bi X}_{A5}$, ${\bi X}_{D5}$, ${\bi X}_{D4}$, ${\bi X}_{D3}$ sub-band matrices are depicted as boxplot in Figs. 3*a*–*h*, respectively. It is evident that the mean values and the standard deviations of the singular value features are different for different EEG classes. The mean values of the first singular value feature of ${\bi X}_{A5}$ sub-band matrix (delta-band features) for silent, noise, music, ANC with noise and ANC with music with noise classes are 4.492, 5.400, 4.234, 3.859 and 3.601, respectively. The mean values of the singular value features of ${\bi X}_{D4}$ sub-band matrix (Alpha-band features) are 7.299, 8.783, 6.476, 4.996 and 5.072 for silent, noise, music, ANC with noise and ANC with noise with music classes. Similar variations are also observed in the singular value features of other sub-band matrices. The alpha-band singular value features show higher differences in the mean value between EEG classes. In silent (with ambient noise), noise and music conditions the subject is able to hear the sound and the situations are unusual. Due to this, the levels of frequency band features ($\delta $, $\theta $, $\alpha $ and $\beta $) remain at a higher level. However, using the ANC headset the noise is reduced by about 20 dB at the ears. This becomes an unusual state of listening and hence may be the features remain always at a lower value. Similarly, when the ANC headset plays a music, the subject listen to the music only without a background noise interference. Therefore, the concentration throughout the listening duration remains intact. This is clearly visible from the standard deviation of the features during ANC with noise and music which is a minimum of all the listening conditions. The $\delta $ band information is uncorrelated with $\theta $, $\alpha $ and $\beta $ bands of the EEG signals which proves that the subjects have not gone to the sleeping stage in any type of listening condition during experiments. The statistical significance of the proposed singular value features of the sub-band matrices of multichannel EEG is performed using the analysis of variance (ANOVA) test. It is evident that all the singular value features from each sub-band matrices have *p*-value <0.001 and these features can be used for classification of the EEG signal.

The sensitivity and the OA values of ELM classifier with three activation functions for five different feature combinations are shown in Table 3. It is evident that, for theta band, alpha band, beta band singular value features and total features cases, the OA values of ELM classifier with ‘radbas’ activation function are higher than other activation functions. The ANC with noise with music class has higher accuracy value as compared to other four classes such as silent, noise, music and ANC with noise. The proposed method has the advantage to capture the $\delta $-wave, $\theta $-wave, $\alpha $-wave and $\beta $-wave information of multichannel EEG data matrix in different sub-band matrices. The singular value features of these sub-band matrices are effective for classification of multichannel EEG signals. In addition to this the overall accuracy between music and ANC with noise and music becomes 83.22%. In this case, the classifier uses total features and ‘radbas’ activation function. ANC is a technique by which the external noise is reduced so that quieter environment can be created or enhances the clarity of music while listening [28]. From the EEG analysis, it is found that classification sensitivity is higher when the ANC system is applied compared to the without ANC system. The application that is suggested is to use ANC headsets while making music to listen during music therapy so that the music can be better perceived by the brain. However, the work proposed in this Letter has future application in music therapy. In music therapy [37], certain specific music is made to listen to the patients to cure diseases. However, due to external background noise, the person listens to music with lesser clarity. Therefore, the effect of music therapy would be suboptimal when there is background noise. However, if we use ANC to reduce background noise, the clarity of music is enhanced and it will have better impact on human brain and health.

**Table 3:** Sensitivity values of each class and OA of ELM classifier with ‘sine’, ‘radbas’ and ‘sigmoid’ as activation functions

Feature selection	Activation function	Silent	Noise	Music	ANC with noise	ANC with noise and music	OA, %
delta band singular values	Sine	67.71	76.95	68.41	76.17	98.61	77.57
	Sigmoid	67.29	73.75	60.63	64.33	97.64	72.72
	Radbas	68.86	76.56	68.57	74.33	99.31	77.52
theta band singular values	Sine	67.57	75.47	67.3	73.83	98.89	76.61
	Sigmoid	67.71	74.84	58.57	62.50	97.92	72.30
	Radbas	69.14	74.84	67.14	76.50	99.17	77.35
alpha band singular values	Sine	68.43	75.94	69.21	75.50	98.75	77.56
	Sigmoid	67.29	74.84	61.27	63.50	98.06	72.99
	Radbas	70.29	76.56	69.52	77.67	99.44	78.69
beta band singular values	Sine	66.00	75.16	66.19	74.33	99.03	76.14
	Sigmoid	66.43	73.75	60.63	62.83	97.78	72.28
	Radbas	68.86	76.72	67.62	77.00	99.44	77.92
total features	Sine	67.00	76.56	66.19	74.33	99.03	76.62
	Sigmoid	66.71	72.66	61.43	63.17	97.92	72.37
	Radbas	68.57	77.03	67.14	75.83	99.31	77.57

## 4 Conclusion

In this Letter, we have investigated the effect of ANC mechanism on multichannel EEG signal. The proposed investigation is performed based on the analysis of multichannel EEG features and classification. The singular value features of the sub-band matrices of multichannel EEG are evaluated. These features are classified using the ELM classifier into silent, noise, music, ANC with noise and ANC with noise with music classes. The ANC with noise with music class has higher sensitivity value than other classes. The important observation from this study is that the ANC reduces the background noise and has a different effect on brain and hence multichannel EEG signals are classified with higher sensitivity. Since the ANC reduces external noise, the music is clearly audible and brain accepts it in a different way. Therefore, for music therapy ANC handsets can be used. In future, the effect of ANC during music therapy can be studied through EEG signal processing.

## 5 Funding and declaration of interests

This work was funded by Council of Scientific of Industrial Research (CSIR) under project no. YSP-3/2013. S. Bagha acknowledges INAE and AICTE, Govt. of India for awarding Teacher Research Fellowship to carry out this work at CSIR-IMMT. Ethical clearance was obtained from Institutional Ethics Committee of AIIMS Bhubaneswar. Conflict of interest: none declared.
